# “gnparser”: a powerful parser for scientific names based on Parsing Expression Grammar

**DOI:** 10.1186/s12859-017-1663-3

**Published:** 2017-05-26

**Authors:** Dmitry Y. Mozzherin, Alexander A. Myltsev, David J. Patterson

**Affiliations:** 1 University of Illinois, Illinois Natural History Survey, Species File Group, 1816 South Oak St., Champaign, 61820 IL USA; 2 IP Myltsev, Kaslinskaya St., Chelyabinsk, 454084 Russia; 3 University of Sydney, Sydney, Australia

**Keywords:** Biodiversity, Biodiversity informatics, Scientific name, Parser, Semantic parser, Names-based cyberinfrastructure, Scala, Parsing Expression Grammar

## Abstract

**Background:**

Scientific names in biology act as universal links. They allow us to cross-reference information about organisms globally. However variations in spelling of scientific names greatly diminish their ability to interconnect data. Such variations may include abbreviations, annotations, misspellings, etc. Authorship is a part of a scientific name and may also differ significantly. To match all possible variations of a name we need to divide them into their elements and classify each element according to its role. We refer to this as ‘parsing’ the name. Parsing categorizes name’s elements into those that are stable and those that are prone to change. Names are matched first by combining them according to their stable elements. Matches are then refined by examining their varying elements. This two stage process dramatically improves the number and quality of matches. It is especially useful for the automatic data exchange within the context of “Big Data” in biology.

**Results:**

We introduce Global Names Parser (*gnparser*). It is a Java tool written in Scala language (a language for Java Virtual Machine) to parse scientific names. It is based on a Parsing Expression Grammar. The parser can be applied to scientific names of any complexity. It assigns a semantic meaning (such as genus name, species epithet, rank, year of publication, authorship, annotations, etc.) to all elements of a name. It is able to work with nested structures as in the names of hybrids. *gnparser* performs with ≈99*%* accuracy and processes 30 million name-strings/hour per CPU thread. The *gnparser* library is compatible with Scala, Java, R, Jython, and JRuby. The parser can be used as a command line application, as a socket server, a web-app or as a RESTful HTTP-service. It is released under an Open source MIT license.

**Conclusions:**

Global Names Parser (*gnparser*) is a fast, high precision tool for biodiversity informaticians and biologists working with large numbers of scientific names. It can replace expensive and error-prone manual parsing and standardization of scientific names in many situations, and can quickly enhance the interoperability of distributed biological information.

**Electronic supplementary material:**

The online version of this article (doi:10.1186/s12859-017-1663-3) contains supplementary material, which is available to authorized users.

## Background

### Conventions

**Fig. 1:**
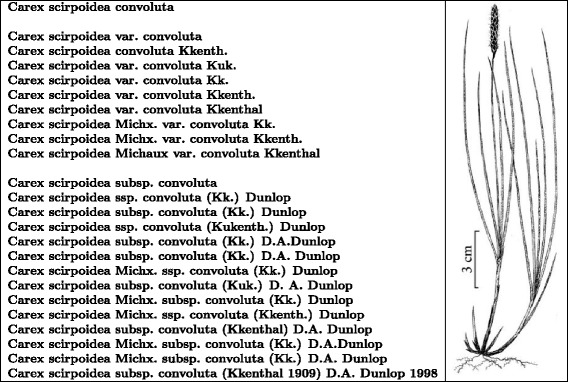
Some legitimate versions of the scientific name for the ‘Northern Bulrush’ or ‘Singlespike Sedge’. The genus (*Carex*), species (*scirpoidea*), and subspecies (*convoluta*) may be annotated (var., subsp., and ssp.) or include or omit the name of the original authority for the infraspecies (Kükenthal), or for the species (Michaux), or for the current infraspecific combination (Dunlop). The name of the authority is sometimes abbreviated, sometimes differently spelled, and may be with or without initials and dates. This list is not complete. Image courtesy of [42]

Throughout the paper we use the terms “name”, “scientific name”, and “name-string” in particular ways. “Name” refers to one or several words that act as a label for a taxon. A “scientific name” is a name formed in compliance with a nomenclatural code (Code) or, if beyond the scope of the Codes, is consistent with the expectations of a Code. The term “name-string” is the sequence of characters (letters, numbers, punctuation, spaces, symbols) that forms the name. A name can be expressed in the form of many name-strings (for example, see Fig. 1). There are about two and a half million currently accepted names for extinct and extant species. There are approximately ten million of legitimately formed scientific names and hundreds of millions of possible name-strings for them. We use the term “elements” for the components of a name-string. Traditionally, in biological literature, scientific names for genera and taxa below genus are presented in *italics*. In this paper, where we wish to emphasize examples of name-strings, we use **bold font**.

### Introduction

Biology is entering a “Big Data” age, where global and fast access to all knowledge is envisaged. Progress towards this vision is still limited in scope. One impediment, especially for the long tail of smaller sources (of which some are not yet digital), is the absence of devices to inter-connect distributed data. The names of organisms are invaluable in “Big Data” biology because they can be treated as metadata and as such can be used to discover, index, organize, and interconnect distributed information about species and other taxa [1]. The use of names for informatics purposes is not straightforward because, for example, there may be many legitimate spellings for a name (Fig. 1). A cyberinfrastructure that uses names to manage information about organisms must determine which name-strings are variant forms of the same scientific name.

Figure 1 presents some of the different legitimate variants of a scientific name in order to make the point that there is not a single correct way to spell scientific names. Because of these variations, fewer than 15% of the names in comparisons of large biological databases could be matched based on exact spellings of name-strings [2]. In order to improve this simple metric for interoperability, we need to identify variants of the same name. We refer to the process of addressing variant spellings (there being other causes of different names for the same taxon) as “lexical reconciliation”. Lexical reconciliation involves linking the alternative spelling variants for the same taxon into a “lexical group”. Most biologists do this intuitively — they recognize that the name-strings in Fig. 1 refer to the same taxon. They do so by “parsing” the name-strings into elements (genus name, species name, authors, ranks etc.) and mentally discarding less significant elements such as annotations and authorship. It then becomes clear all of name-strings are formed around the Latin elements **Carex scirpoidea convoluta**. We refer to the form of the scientific name without authority or annotations as the “canonical form”. Further analysis of the name-strings reveals two different lexical groups (separated in Fig. 1 by a line break) for, probably, one taxonomic concept:

- **Carex scirpoidea var. convoluta** description by **Kükenthal**
- **Carex scirpoidea subsp. convoluta** rank determination by **Dunlop**.

In the past, the need to parse scientific names to form normalized names has mostly been achieved manually. A person familiar with rules of botanical nomenclature would be able to analyse the 24 name-strings in this example with relative ease, but not thousands or millions of name-strings - especially if they include scientific names to which more than one nomenclatural code may be applied. The manual splitting of names into even only two parts — the latinized elements of taxon names that make up the canonical form and the authorship — is slow and therefore expensive. To scale this exercise up requires an algorithmic solution, a scientific name parser!

The strategy of the algorithmic approach is to identify which combinations of the most atomic parts of a name-string (i.e. the UTF-8 encoded characters) represent words (such as genus name, species name, authors, annotations) or dates. An early algorithmic approach to parsing scientific names was with “regular language” implemented as regular expression [3]. A regular expression is a sequence of characters that describes a search pattern [4]. For example, a regular expression “[A-Z][a-z]{2}” recognizes a word that starts from a capital letter followed by two small letters (e.g. “Zoo”). Scientific names almost universally follow patterns that are influenced by the Codes of Nomenclature: such as the use of spaces to separate words, capitalization of generic names and authors, or the inclusion of four digit dates between the middle of the 18th century and the present. This makes most names amenable to parsing by regular expressions. Current examples of scientific name parsers based on regular expressions are GBIF’s *name-parser* [5], and *YASMEEN* [6].

While regular expression is a powerful approach to string parsing, it has limitations. It cannot elegantly deal with name-strings where an authorship element is present in the middle of the name (for example **Carex scirpoidea Michx. subsp. convoluta (Kük.) D.A.Dunlop**). Indeed, regular expressions are not well suited to any targets with recursive (nested) elements [7], such as hybrid formulae (e.g. **Brassica oleracea L. subsp. capitata (L.) DC. convar. fruticosa (Metzg.) Alef. × B. oleracea L. subsp. capitata (L.) var. costata DC.**). Name parsing built on regular expressions is impractical for complex name-strings.

Another limitation with most regular expression software tools is that they are “black boxes” that allow developers very limited interaction with the parsing process. They do not reveal much information about the parsing context and developers cannot call a procedure during a parsing event. As a result, complex regular expression-based parsers are difficult to implement and maintain, and functions such as error recovery, detailed warnings, descriptions of errors are missing.

We wanted to deal with scientific names across a very broad range of complexity and to give more flexibility than can be achieved with a regular expression approach. We believe that a scientific name parser should satisfy the following requirements.

1. **High Quality.** A parser should be able to break names into their semantic elements to the same standards that can be achieved by a trained nomenclaturalist or better. This will give users confidence in the automated process and allow them to set aside tedious and expensive manual parsing.
2. **Global Scope.** A parser should be able to parse all types of scientific names, inclusive of the most complex name-strings such as hybrid formulae, multi-infraspecific names, names with multilevel authorships and so on. No name-strings should be left unparsed, otherwise biological information attached to them may remain undiscoverable.
3. **Parsing Completeness.** All information included in a name-string is important, not just the canonical form of the scientific name. Authorship, year, rank information allow us to distinguish homonyms, similar names, synonyms, spelling mistakes, or chresonyms. Access to such information improves the performance of subsequent reconciliation (the mapping of all alternative name-strings for the same taxon against each other).
4. **Speed.** Users, especially large-scale aggregators of biodiversity data, are more satisfied with speedy processing of data as it allows them to move forward to more purposeful value-adding tasks. Speed reduces the purchasing/operating costs of the hardware used for production parsing.
5. **Accessibility.** To be available to the widest possible audience, a parser should be released as a stand-alone program, have good documentation, be able to work as a library, to function as a command line tool, as a tool within a graphical interface, to run as a socket or as RESTful services.

These requirements became our design goals. Based on our experience with prototype systems, we chose to use Parsing Expression Grammar and Scala language.

### Adoption of Parsing Expression Grammar

Parsing Expression Grammar (PEG) [8] have been introduced for parsing strings. PEG allows developers to define the rules (“grammar”) that describe the general structure of target strings. Such rules can be used to deconstruct scientific names. The rules are built from the ground up, starting from the simplest — such as a combination of “characters” separated by “spaces”. That ‘rule’ identifies most “words”. Digits and other characters make dates identifiable. Further rules can be applied, such as a “genus” rule can describe a part of a polynomial name-string in which the first word begins with combination of a “capital_character” followed by several “lower_case_characters” that fall within a relatively small spectrum of allowed characters; “authorship” would consist of one or more capitalized words and followed perhaps by a “year”. Within some instances of authorship, authors may be grouped to form “author-teams”. PEG rules are designed to be recursive. They can be expanded to deal with increasingly complex name-strings, or address errors such as absent or extra spaces, or OCR errors. Each rule can have programmatic logic attached, making the PEG approach very flexible. We believe that PEG suits our goals better than regular expressions for the following reasons:

- PEG is better suited than regular expressions for strings with a recursive structure;
- the syntax of scientific names is formal enough to be closer to an algebraic structure rather than to a natural language. Inconsistencies and ambiguities in scientific name-strings are relatively rare because they usually comply with the requirements and conventions of nomenclatural codes;
- scientific name-strings are short enough to avoid problems with computational complexity and memory consumption;
- programming a parser with PEG can describe parsing rules in a domain-specific language;
- domain-specific languages offer great flexibility for logic within the rules, for example to report errors in name-strings.

The Global Names project created a specialized parsing library *biodiversity* in 2008 [9]. It was written in Ruby and based on PEG. It uses the *TreeTop* Ruby library [10] as the underlying PEG implementation.

The PEG approach allowed us to deal with complex scientific names gracefully. It gave us flexibility to incorporate edge cases and to detect common mistakes during the parsing process. The *biodiversity* library has enjoyed considerable popularity. At the time of writing, it had been downloaded more than 150,000 times [11], it is used by many taxon name resolution projects (e.g. Encyclopedia of Life [12], Canadian Register of Marine Species (CARMS) [13], the iPlant TNRS [14], and World Registry of Marine Species (WoRMS) [15]. According to statistics compiled by BioRuby, *biodiversity*, at the time of writing, has been the most popular bio-library in the Ruby language [16].

We were pleased with PEG approach for parsing scientific names, but regard the *biodiversity* parser library as a working prototype. It has allowed us to make further improvements and deliver a better, faster production-grade parser.

### Other approaches

There is a growing number of algorithms and tools in machine learning and natural language processing that aim to recognize parts of texts. They include statistical parsing [17], context-free grammars [18], fuzzy context-free grammars [19], and named entity recognition [20]. Unsupervised deep learning [21, 22] increases the quality of entity recognition without extensive curation and programming efforts by people. We chose not to use these approaches for the following reasons.

- The limited scope of a parser. A parser of scientific names very rarely needs to work with name-strings of more than 15 words.
- There is no need for recognition. A scientific name-string parser is usually applied to preexisting lists of scientific names. There is no requirement to recognize scientific names in larger bodies of text. Other scientific name recognition and discovery tools are available.
- Formal grammar. Scientific names are formed in compliance with well-defined and formal codes of nomenclature. They have predictable structures making the requirements for a scientific name-string parser to be more similar to parsers of programming languages than to tools designed to work with natural languages.
- Scale and throughput. We created the parser to serve the needs of biodiversity aggregators. A core design requirement was to develop a lightweight library for inputs of millions of scientific name-strings per second, and to be processed locally.
- Stand-alone approach. We did not wish the parser to rely on local or remote previously known information of genera, species, author names, or other scientific names. *gnparser* relies instead on morphological features of scientific name-strings.
- Determinism. Biologists know that there is only a single correct parsed version of a scientific name. A scientific names parser must produce a single “correct” result for each input string. A parser should provide meta information on every part of the string.

### Adoption of Scala

The pre-existing *biodiversity* package is not speedy and cannot scale because it uses Ruby as its programming language. Ruby is one of the best languages for rapid prototyping, but it is an interpreted dynamic language with, originally, a single-threaded runtime during execution. This makes it slow and inappropriate for “Big Data” tasks. We concluded that we needed a replacement language environment with the following properties:

- a mature technology;
- multithreaded, with high performance and scalability;
- an active support community with an Open source friendly culture;
- a wide range of libraries: utilities, web frameworks, etc.;
- a powerful development environment with IDEs, testing frameworks, debuggers, profilers and the like;
- mature libraries for search and cluster computations;
- interoperable with languages popular in scientific community (R, Python, Matlab);
- natural support of domain specific languages embedded in the hosted language.

While many of the properties are true for Ruby, other properties, such as high performance, scalability and interoperability, are not. To meet all requirements, and exploiting what we had learned from *biodiversity*, we rewrote the code using Scala (a Java virtual machine programming language [23]), and the Open source *parboiled2* library [24] which we improved [25]. The *parboiled2* library implements PEG in Scala. An alternative to *parboiled2* is the Scala combinators library [26]. We did not use it because it is slow and has memory consumption problems.

The functional programming features of Scala allowed us to build a domain specific language that describes the grammar’s rules to parse scientific names. This produces a Parsing Expression Grammar with considerably more flexibility than external lexers such as Bison or Yacc. As this domain specific language is within *parboiled2*, it can take advantage of the Macro capacity of Scala [27] to optimize the compilation of the code and the subsequent running of the program. As a result, the software performs with high efficiency. The resulting *gnparser* library is faster, more scalable and more flexible than its predecessor.

We limited this version to work with scientific names that comply with the botanical, zoological, and prokaryotic codes of nomenclature, but not with names of viruses because they are formed in different ways [2, 28] and need a different PEG. We intend to add this later.

## Implementation

The *gnparser* project is entirely written in Scala. It supports two major Scala versions: 2.10.6+ and 2.11.x. The code is organized into four modules:

1. “*parser*” is the core module used by all other modules. It parses scientific names from the most atomic components of a name-string to semantically-defined terms. It includes the parsing grammar, an abstract syntax tree (AST) composed of the elements of scientific names, warning and error facilities. When the parsing is complete and semantic elements of name-strings have been assigned to AST nodes, the elements can be recombined and formatted to meet further needs. For example:
*normalizer* converts input name-strings into a consistent style;
*canonizer* creates canonical forms of the latinized elements of names;
*JSON renderer*, the parsing result is converted to JSON [29] to allow developers to work with the output using other languages. The output (Fig. 2, also see Results and discussion) has the following information: **’details’** contains the JSON-representation of a parsed scientific name; **’quality_warnings’** describes potential problems if names are not well-formed; **’quality’** depicts a quality level of the parsed name; and **’positions’** maps the positions of every element in a parsed name to the semantic meaning of the element. Full and formal explanation of all parser fields is given as a JSON schema and can be found online [30] [also see Additional file 1].

**Fig. 2:**
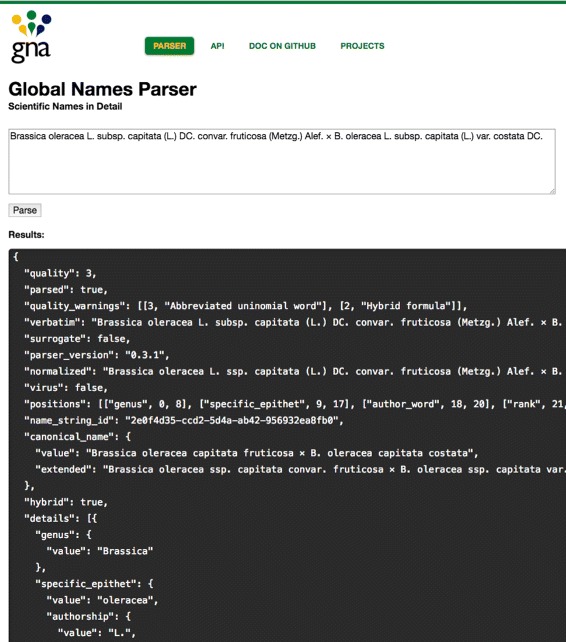
Web Graphical User Interface [43]. In this example a user entered a name-string of a hybrid name consisted of 21 elements. The “Results and discussion” section contains detailed parsed output using compact JSON format
2. The “*spark-python*” module contains facilities to use “*gnparser*” with Apache Spark scripts written in Python. Apache Spark is a highly distributive and scalable development environment for processing massive sets of data. Spark is written in Scala, but can also be used with Python, R and Java languages. Spark programs written in Java and Scala are able to run “*parser*” in a distributed fashion natively.
3. The “*examples*” module contains examples to assist developers in adding “*parser*” functionality into other popular programming languages such as Java, Scala, Jython, JRuby, and R.
4. The “*runner*” module contains the code that allows users to run “*parser*” from a command line as a standalone tool or to run it as a TCP/IP socket or HTTP web server. It depends on the “*parser*” module. The core part is the launch script “*gnparse*” (for Linux/Mac and Windows) that creates a JVM instance and runs “*parser*” on multiple threads against the input provided via a socket or file. This module also contains a web application and a RESTful interface to offer simpler ways to access “*parser*”. “*web*” achieves interactions with “*parser*” via HTTP protocol. It works both with simple web (HTML) and REST API interfaces. Figure 2 illustrates a parsing example using the web-interface. Socket and REST services use Akka framework which makes them highly concurrent and scalable.

“*parser*“ and “*examples*“ can run in JVM 1.6+. “*runner*” requires JVM 1.8+. Documentation is available in a README file [see Additional file 2].

### Parsing rules

*gnparser* v0.3.1 contains 76 PEG rules. In turn, these rules make use of more elementary rules provided by the *parboiled2* library. The rules are domain-specific based on hours of conversations with leading taxonomists, study of nomenclatural codes, and feedback of the users.

As an example, the *yearNumber* rule is given below. It detects the year in which a name was published. *Rule[Year]* is a type of the returning value of the rule. Using domain-specific language and elementary rules of *parboiled2* we capture the start and the end positions of a year substring (lines #1 and #2). This matches a substring that represents a year in scientific name-strings. A publication year is usually a number between 1753 [31] and the present. A year substring might have one or two digits substituted with question marks if the exact year of a publication is unknown. The capture is then passed as a parameter to a parser action (line #3). Parser action, a Scala function, might produce warnings or a class instance of defined type (*Rule[Year]*).



We then assemble more complex inter-dependent rules (lines #5 to #10), and finally combine all of them into the rule *year* on line #11 that consists of prioritized alternatives of all previously defined rules.



This enables the incorporation of the *year* rule into all cases where it might be needed. For example on line #12 we indicate that *year* must be present in the matcher for the *authorsYear* rule.



### Installation

“*gnparser*” is available for launch in three bundles.

- A *parser* artifact is provided via the Maven central repository of Java code [32]. Physically it is a relatively small jar file without embedded external dependencies. The artifact can be accessed in custom projects by a build system such as Maven, Gradle, or SBT. The build system identifies and provides access to all dependent jars.
- A Zip-archived “fat jar” is located at the project’s GitHub repository. The jar contains the compiled files of *gnparser* along with all necessary dependencies to launch it within JVM. The archive is also bundled with a launch script (for Windows, OS X and Linux) that can run a command line interface to *gnparser*.
- The project’s Docker container image is located at Docker Hub [33]. Docker provides an additional layer of abstraction and automation of operating-system-level virtualization on Linux. It can be thought of as a lightweight virtualization technology within a Linux OS host. When it is setup properly, everything — starting from JVM and ending with Scala and SBT — can be run with simple commands that will, for example, pull the *gnparser*’s Docker image from the DockerHub, and run the socket or web server on an appropriate port.

### Testing methods

Data for our tests were sets of 1000 and 100,000 name-strings randomly chosen from 24 million unique name-strings of the Global Names Index (GNI) [34]. The name-strings in GNI are collected from a large variety of biodiversity data sources and are pre-identified as scientific names. While GNI contains some incorrectly classified strings, it is the largest compilation of name-strings representing scientific names. It is not biased towards any particular taxon or particular variant of name, and so the extracted datasets are believed to represent naturally occurring data quite well. The datasets are randomly chosen and are therefore mixtures of well-formed names, lexical variants of names, names with formatting and spelling mistakes, and name-strings that were misrepresented as names. Name-strings in the sets are independent of each other. An evaluation dataset with 1000 names is included as Additional file 3.

We compared the performance of *gnparser* with two other projects: *biodiversity* parser [9, 35] (also developed by Global Names team), and the GBIF *name-parser* [5]. The following versions were used: *gnparser* v. 0.2.0, GBIF *name-parser* v. 0.1.0, *biodiversity* v. 3.4.1. To make comparisons, we calculated *Precision*, *Recall* and *Accuracy* (as described below) using a dataset consisting of 1000 name-strings. We also tested the YASMEEN parser from iMarine [6]. With our dataset, YASMEEN generated many more mistakes than other parsers (*Precision* 0.534, *Recall* 1.0, *F*1 0.6962), and was unable to finish a full dataset without crashing. We excluded it from further tests.

To estimate the quality of the parsers, we relied on their performance in representing canonical forms and terminal authorships. A canonical form represents the latinized elements of taxon names, while the terminal authorship refers to the author of the lowest subtaxon found in the scientific name. For example, with **Oriastrum lycopodioides Wedd. var. glabriusculum Reiche**, the canonical form is **Oriastrum lycopodioides glabriusculum** and the terminal authorship is **Reiche**, not **Wedd**.

When both the canonical form and the terminal authorship were determined correctly we marked the result as true positive (*N*_*tp*_). If one or both of them were determined incorrectly, the result was marked as a false positive (*N*_*fp*_). Name-strings correctly discarded from parsing were marked as true negatives (*N*_*tn*_). False negatives (*N*_*fn*_) were name-strings which should have been parsed, but were not. The results of the tests are summarized in Table 1:

**Table 1:** Precision/Recall for parsers applied to 1000 name-strings

	gnparser	gbif-parser	Biodiversity
*True positive*	978	955	971
*True negative*	13	12	13
*False positive*	9	32	16
*False positive*	0	1	0
*Precision*	0.989	0.968	0.984
*Recall*	1.0	0.999	1.0
*F1*	0.994	0.983	0.992
*Accuracy*	0.989	0.967	0.984

*Accuracy* — the proportion of all results that were correct. It is calculated as:

$$Accuracy = \frac{N_{tp} + N_{tn}}{N_{tp} + N_{tn} + N_{fp} + N_{fn}} $$

*Precision* — the proportion of name-strings parsed correctly compared to all detected name-strings. It is calculated as:

$$Precision = \frac{N_{tp}}{N_{tp} + N_{fp}} $$

*Recall* — the proportion of correctly detected name-strings relative to all parseable name-strings and is calculated as:

$$Recall = \frac{N_{tp}}{N_{tp} + N_{fn}} $$

The *F*1−*measure* is a balanced harmonic mean (where *Precision* and *Recall* have the same weight). When *Precision* and *Recall* differ, *F*1−*measure* allows results to be compared. It is calculated as

$$F1 = \frac{2 \times Precision \times Recall}{Precision + Recall} $$

Some names in the dataset were not well-formed. If a human could extract the canonical form and the terminal authorship from them, we included them in our assessment. Examples of such name-strings are **“Hieracium nobile subsp. perclusum (Arv. -Touv.) O. Bolòs & Vigo”** (the problem for the parser here is an introduced space within an author’s name), **“Campylium gollanii C. M?ller ex Vohra 1970 [1972]”** (with a miscoded UTF-8 symbol and an additional year in square brackets), **“Myosorex muricauda (Miller, 1900).”** (with a period after the authorship).

Parsers analyze the structure of name-strings, but they cannot determine if a string is a “real” name. For example, in the case of a name-string that has the same form as a subspecies such as **“Example name Word var. something Capitalized Words, 1900”**. In such a case, the identification of a canonical form as **“Example name something”** and terminal authorship as **“Capitalized Words, 1900”** would be considered a true positive. Clearly, it will be important for name-management services to distinguish between name-strings of scientific names, names of viruses, surrogate names, and non-names. To find out how well parsers distinguished strings which are not scientific names, we calculated *Accuracy* for discarded/non-parsed strings. If the parser worked well, non-parsed strings would include only names of viruses and terms that do not comply with the codes of zoological, prokaryotic, and botanical nomenclature.

We processed 100,000 name-strings with each parser. Each parser discarded close to 1,000 name-strings as non-parseable. *Accuracy*, in this case, provided the percentage of correctly discarded names out of all discarded by the parser names. We do not know *Recall*, as it was not reasonable to manually determine this for 100,000 names. To get a sense of names which should be discarded but were parsed instead, we analysed intersections and differences of the results between the three parsers as shown in Table 2.

**Table 2:** Accuracy of non-parseable names detection out of 100,000 name-strings

	gnparser	gbif-parser	Biodiversity
*True discarded*	1131	1082	1161
*Correctly discarded*	1129	940	1152
*Incorrectly discarded*	2	142	9
*Accuracy*	0.998	0.869	0.992

To establish the throughput of parsing we used a computer with an Intel i7-4930K CPU (6 cores, 12 threads, at 3.4 GHz), 64GB of memory, and 250GB Samsung 840 EVO SSD, running Ubuntu version 14.04. Throughput was determined by processing 1,000,000 random name-strings from Global Names database.

To study the effects of parallel execution on throughput we used the *ParallelParser* class from *biodiversity* parser. We used ‘*gnparse file –simple*’ (a command line-based script set to return simplified output) for *gnparser*. For GBIF *name-parser*, we created a thin wrapper with multithreaded capabilities [36]. The following versions had been used for throughput benchmarks: *gnparser* v. 0.3.1, GBIF *name-parser* v. 0.1.0, *biodiversity* v. 3.4.1.

## Results and discussion

We discuss and compare *gnparser*, GBIF *name-parser* and *biodiversity* parser in the context of our requirements for quality, global scope, parsing completeness, speed, and accessibility.

### High quality parsing

Quality is the most important of the 5 requirements. GBIF *name-parser* uses regular expressions approach, while *gnparser* and *biodiversity* parsers use the PEG approach. Results for quality measurements are shown in Tables 1 and 2. We include the 1,000 tested names as Additional file 3.

If test data contain a large proportion of true negatives (*N*_*tn*_) *Accuracy* will not be a good measure as it favors algorithms that distinguish negative results rather than finding positive ones. We manually checked our test datasets and established that ≈1*%* were not scientific names. Given that true negatives are rare, they will have very limited influence on *Accuracy*. *Recall* for all parsers was high, hence false negatives are not important.

*Accuracy* is probably the best measure for our tests. All 3 parsers performed very well, with *Accuracy* values higher than 95%. Both *gnparser* and *biodiversity* parser approached the 99% mark which we regard as the metric for production quality. Most of the false positives came from name-strings with mistakes. For example, out of 11 false positives (below) that *gnparser* found in the 1000 name-string test data set, only 2 (the first 2) were well-formed names.

- **Eucalyptus subser. Regulares Brooker**
- **Jacquemontia spiciflora (Choisy) Hall. fil.**
- **Acanthocephala declivis variety guianensis Osborn, 1904**
- **Atysa (?) frontalis**
- **Bumetopia (bumetopia) quadripunctata Breuning, 1950**
- **Cyclotella kã** ^**1**^ **/**_**4**_ **tzingiana Thwaites**
- **Elaphidion (romaleum) tæniatum Leconte, 1873**
- **Hieracium nobile subsp. perclusum (Arv. -Touv.) O. Bolòs & Vigo**
- **Leptomitus vitreus (Roth) Agardh?**
- **Myosorex muricauda (Miller, 1900).**
- **Papillaria amblyacis (M** <**81** >**ll.Hal.) A.Jaeger**

We do expect a parser to deal with names that are not well-formed. That means overcoming problems such as aberrant characters which might arise from Unicode character miscodings, inappropriate annotations, or other mistakes. To alert users, *gnparser* generates a warning when it identifies a problem in a name-string. The other parsers do not have this feature.

When parsers reach ≈80*%* *Accuracy*, they hit a “long tail” of problems where each particular type of a problem is rare. Every new manual check of additional test sets of 1,000–10,000 name-strings reveals new issues. Examples of these challenges are given elsewhere [2]. For all three parsers, developers have to perform the meticulous task of adding new rules to address each rare case. That is, parsers need to be subject to continuous improvement. The problems found during preparation of this paper are being addressed in the next version of *gnparser*. As the parsing rules improve, we believe that *gnparser* can reach >99.5*%* *Accuracy* without diminishing *Recall*.

As we incorporate new rules to increase *Recall*, we have to consider the risks of reducing *Precision* by introducing new false positives. For example, the GBIF *name-parser* allows the genus element of a name-string to start with a lowercase character. As a result the name-strings below were parsed as if they were scientific names, while the other parsers ignored them:

- **acid mine drainage metagenome**
- **agricultural soil bacterium CRS5639T18-1**
- **agricultural soil bacterium SC-I-8**
- **algal symbiont of Cladonia variegata MN075**
- **alpha proteobacterium AP-24**
- **anaerobic bacterium ANA No.5**
- **anoxygenic photosynthetic bacterium G16**
- **archaeon enrichment culture clone AOM-SR-A23**
- **bacterium endosymbiont of Plateumaris fulvipes**
- **bacterium enrichment culture DGGE band 61_3_FG_L**
- **barley rhizosphere bacterium JJ-220**
- **bovine rumen bacterium niuO17**

Strategies like these may increase *Recall* with certain low-quality datasets, but they decrease *Precision*. Many “dirty” datasets contain recurring problems. As an example, DRYAD contains many name-strings in which elements of scientific names are concatenated with an interpolated character such as ‘_’ (e.g. “Homo_sapiens” and “Pinoyscincus_jagori_grandis”) [2]. For them, our solution was to include a “preparser” script which “normalizes” known problems that are inherent within particular datasets and then apply a high quality parser to the result.

Our testing also revealed differences between regular expressions and PEG approaches. Both can achieve high quality results with canonical forms of scientific names, but the regular expressions are less suitable for more complex name-strings. The recursive or nested nature of some scientific names can cause problems which become insurmountable for regular expressions.

### Global scope

If we want to connect biological data using scientific names, no name-strings should be missed or rejected, no matter how complex they are. During our testing we found that *Accuracy* of GBIF’s *name-parser* was depressed because, in part,the parser did not recognize hybrid formulae and infrasubspecific names with more then one infraspecific epithet. This case underscores the limitations of the regular expression approach. As examples, the following were not parsed by the GBIF *name-parser*:

**Erigeron peregrinus ssp.callianthemus var. eucallianthemus** (a name-string with two infraspecificx epithets)

**Polyporus varius var. nummularius f. undulatus (Pilát) Domanski, Orlos & Skirg.** (two infraspecific epithets)

**Salvelinus fontinalis x Salmo gairdneri** (hybrid formula)

**Echinocereus fasciculatus var. bonkerae × E. fasciculatus var. fasciculatus** (hybrid formula)

The PEG approach supports nested parsing rules to create progressively more complex rules that manage such cases. The capacity to address recursion allows *gnparser* to handle the full spectrum of scientific names that we have presented to it.



### Parsing Completeness

The extraction of canonical forms from name-strings representing scientific names is the most beneficial and widely used parsing goal. Sometimes, however, this may not be sufficient because the canonical form does not always distinguish a name completely.

In the example in Fig. 1 **Carex scirpoidea convoluta** is a canonical form for **Carex scirpoidea var. convoluta Kükenthal** and **Carex scirpoidea ssp. convoluta (Kük.) Dunlop.** The first non-parsed name-string refers to the variety **convoluta** of **Carex scirpoidea** that had been described by **Kükenthal**. The second captures Dunlop’s reclassification of **convoluta** as a subspecies. We are not able to distinguish between these two different names without knowing the rank and/or the corresponding authorship. Furthermore, it is useful to see in the second example that **(Kük.)** was the original author and **Dunlop** was the author of the new combination. Also, canonical forms do not distinguish between homonyms. The heather, *Pieris japonica* (Thunb.) D. Don ex G. Don and the butterfly, *Pieris japonica* Shirôzu, 1952 have the same canonical form **Pieris japonica**.

After matching by canonical form, rank, authors, and “types” of authorship allow us to distinguish name-strings with similar or identical canonical elements. The name-string **Carex scirpoidea Michx. var. convoluta Kükenth.** adds the information that the species **Carex scirpoidea** was described by **Michx** but is not evident in the examples in the paragraph above.

Another area in which parsers with limited abilities can give misleading results is with negated names [2]. In these cases, the name-string includes some annotation or marks to indicate that the information associated with the name does NOT refer to the taxon with the scientific name that is included. Examples include **Gambierodiscus aff toxicus** or **Russula xerampelina-like sp**.

All components of a name may be important and need to be parsed and categorized. With *gnparser*, we describe the meaning of every element in the parsed name-string and present the results in JSON format. Parsing of **Carex scirpoidea Michx. subsp. convoluta (Kük.) D.A. Dunlop** gives the following JSON output

The output includes the semantic meaning of all parsed elements in a name-string, indicates if the name-string was parsed successfully, if it is a virus name, a hybrid, or a surrogate. Surrogates are name-strings that are alternatives to names (such as acronyms) and they may or may not include part of a scientific or colloquial name (e.g. **Coleoptera sp. BOLD:AAV0432**). The output also includes a statement of the position of each element in the name-string. Last, but not least, the JSON output contains UUID version 5 calculated from the verbatim name-string. This UUID is guaranteed to be the same for the same name-string, promoting its use to globally connect information and annotations.

The output usually covers every semantic element in the name-string. The fields in the output illustrated above have the following meanings.

- **name_string_id:** UUID v5 identifier;
- **parsed:** whether a name-string was successfully parsed (true/false);
- **quality:** how well-formed a name-string is (range from 1 to 3, 1 is the best);
- **parser_version:** version of a parser used;
- **verbatim:** name-string as was submitted to *gnparser*;
- **normalized:** name-string modified by the parser to give a normalized style;
- **canonical_name:** a special form of normalization that includes only the scientific elements of the name, this form is contained within most name-strings relating to scientific names;
- **hybrid:** whether the name-string refers to a hybrid (true/false);
- **surrogate:** whether a name-string is a surrogate name (true/false);
- **details:** describes the semantic elements within the name-string inclusive of the following;
- **genus:** reports the genus part of the name (in this case Carex);
- **specific epithet:** reports the species epithet (scirpoidea);
- **authorship:** reports the authorship of the combination (Michx.);
- **basionym authorship:** reports the authorship of the basionym (Michx.)
- **infraspecific epithets:** reports the infraspecies name if present (convoluta) with rank (ssp.)
- **authorship:** reports the authors of the infraspecies name ((Kük.) D. A. Dunlop)
- **basionym authorship:** reports the author of the basionym of infraspecies name element ([“Kük.”]);
- **combination authorship:** reports the author of the infraspecies name combination (D. A. Dunlop); and
- **positions:** identifies each name element and where it starts and ends.

The complete list of fields for the *gnparser*’s output exists as a JSON Schema file [30] [see Additional file 1].

### Parsing speed

In the areas of performance discussed above, there is little difference between *biodiversity* parser and *gnparser*. There is, however, a dramatic difference in their parsing speed and ability to scale. Parsing tasks that took 20 hours with earlier *biodiversity* parsers can now be completed in a few minutes on a multithreaded computer. Parsing is a key to other services such as name-reconciliation and subsequent resolution. Improvements to the speed of the parser will increase user satisfaction elsewhere.

**Fig. 3:**
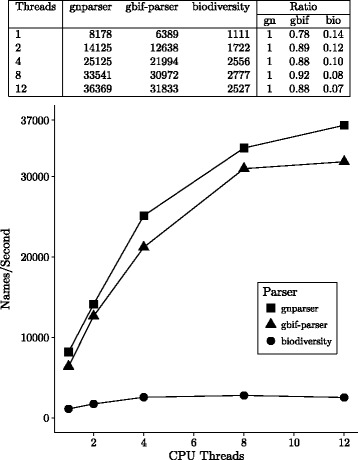
Names parsed per second by GN, GBIF and Biodiversity parsers (running on 1–12 parallel threads)

Results on the speed performance are given in Fig. 3. The performance depends on the number of CPU threads used. On 1 thread *gnparser* was 7 times faster than *biodiversity*, 10 times faster on 4 threads, and 14 times faster on 12 threads.

*gnparser* displays functionality not presented in the GBIF *name-parser* as described in previous sections. In spite of this additional functionality *gnparser* outperformed other tested parsers.

### Accessibility

By ‘accessibility’ we refer to the ability of the software code to be used by a wide audience. For Open source projects, accessibility is very important. If more people use a software, the more cost-effective is its development.

Parsing scientific names is essential for organizing biodiversity data. Many biodiversity database environments and projects include a parsing algorithm. Examples are uBio [37], the Botanical Society of Britain and Ireland [38], FAT [39], NetiNeti [40], and Taxonome [41]. A modular approach offers an option of re-use and avoids replication of effort. *biodiversity* was the first biodiversity parser to be released as a stand-alone package that could be used as a module — as it was with the iPlant project [35]. The same approach has now been adopted with the GBIF *name-parser* [5], *YASMEEN* [6], and *gnparser*.

We designed *gnparser* with accessibility in mind from the outset. Scala language allows the use of *gnparser* as a library in Scala, Java, Jython, JRuby and a variety of other languages based on Java Virtual Machine it can also be used natively in R and Python via JVM-binding libraries. Apache Spark, a “Big Data” framework, is also supported. The following example illustrates how a client written in Jython can access the *gnparser* functionality.

from org.globalnames.parser import

ScientificNameParser

snp = ScientificNameParser.instance()

result = snp.fromString(~Homo sapiens

L.~).renderCompactJson()

print result

If programmers want to use *gnparser* in some JVM-incompatible language they can connect to the parser via a socket server interface. There is also a command line tool, a web interface, and a RESTful API. In 2016, Encyclopedia of Life started to parse name-strings using *gnparser* socket server.

We pay close attention to documentation, trying to keep it detailed, clear, and up to date. We have an extensive test suite [see Additional file 4] that describes the parser’s behavior and contains examples of *gnparser* functionality and output format.

This commitment to accessibility creates a larger potential audience for the parser, and will help many researchers and programmers deal with the problems that arise from variant forms of scientific names.

## Conclusions

The performance of the scientific names parsers is summarised in Table 3. The two PEG-based parsers — *biodiversity* and *gnparser* are similar. They are based on the same algorithmic approach and follow similar design goals. While we had the option of modifying the rules for *biodiversity* to improve *Accuracy*, we preferred to create a new tool from scratch to overcome limitations in speed, scalability and accessibility. We needed to address speed at Global Names because existing software took too long to parse or reparse 24 million name-strings. *gnparser* can be used natively by larger variety of programming languages than *biodiversity*, because JVM-based languages and tools are so widely used. Our first goal for *gnparser* was complete coverage of the *biodiversity*’s test suite. We continue to improve *gnparser* while *biodiversity* entered maintenance mode. That explains a slight difference in *Accuracy* by these two parsers.

**Table 3:** Summary comparison of Scientific Name Parsers

	gnparser	gbif-parser	Biodiversity
*Accuracy*	98.9%	96.7%	98.4%
*Hybrid formulas support*	Yes	No	Yes
*Infrasubspecies support*	Yes	No	Yes
*Throughput (names/s/thread)*	8178	6389	1111
*Parsing details*	Complete	Partial	Complete
*Library for the same languages*	Yes	Yes	Yes
*Library for other languages*	Yes	Yes	No
*Command line tool*	Yes	No	Yes
*Socket server*	Yes	No	Yes
*Web interface*	Yes	Yes	Yes
*RESTful service*	Yes	Yes	Yes

*gbif-parser* is a high quality product. However, its regular expressions-based algorithm limits its usability. The recursive nature of some scientific names creates significant obstacles for intrinsically non-recursive algorithms such as regular expressions. Coverage of multi-infraspecific names and hybrids, both with recursive patterns, is prohibitively expensive for such an approach.

In conclusion, this paper describes *gnparser*, a powerful tool for working with biodiversity information. It transforms names of taxa into their semantic elements. This allows standardization of names by, for example, representing them as canonical forms. This step dramatically improves name matching within and among data sources, and this increases the amount of data on a single taxon that can be integrated. Parsing can be used to improve the discovery of names in sources, and creating a common taxonomic index to multiple sources. Parsing allows users to extract, compare and analyse metadata within the name-strings, and allowing comparisons of the efforts of individuals or to map trends over time. The *gnparser* tool is released under MIT Open source license, contains command line executable, socket, web, and REST services, and is optimized for use as a library in languages like Scala, Java, R, Jython, JRuby.

## Availability and requirements

**Project Name:** gnparser

**Project home page:** https://github.com/GlobalNamesArchitecture/gnparser

**Operating System:** Any platform able to run JVM 1.8

**Programming Language:** Scala

**License:** The MIT License

**Other requirements:** docker (optional)

**Any restrictions to use by non-academic:** no restriction

The data supporting the conclusions of this article are available in the repository https://github.com/GlobalNamesArchitecture/gnparser-paper under the *data* directory.

## Additional files

Additional file 1 Includes a full and formal explanation of all parser fields as a JSON schema. (JSON 9 kb)

Additional file 2 README.rst file that is converted to HTML format. It is also available at project home page [44]. (ZIP 7 kb)

Additional file 3 1,000 name-strings randomly selected from GNI and used to determine *Accuracy*, *Precision* and *Recall* data (Table 1). (TXT 32 kb)

Additional file 4 Extensive test suite that describes the parser’s behavior. It is also a source of examples of parser functionality and output format. Test suite consists of a pipe delimited input (scientific name) and parsed output in JSON format. (TXT 253 kb)

## Abbreviations

AAM: Alexander A. Myltsev
API: Application program interface
AST: Abstract Syntax Tree
BHL: Biodiversity heritage library
DJP: David J. Patterson
DYM: Dmitry Y. Mozzherin
GBIF: Global Biodiversity information facility
GNA: Global names architecture
GNI: Global names index
JSON: JavaScript object notation
JVM: Java virtual machine
PEG: Parsing expression grammar
REST: Representational state transfer
