# Supplementary material for: “gnparser”: a powerful parser for scientific names based on Parsing Expression Grammar
Source: BMC Bioinformatics. 2017 May 26;18:279. doi: 10.1186/s12859-017-1663-3 (PMC5446698; doi:10.1186/s12859-017-1663-3)
Supplement: Supplementary file 2 — README.rst file that is converted to HTML format. It is also available at project home page [44]. (ZIP 7 kb) [file 12859_2017_1663_MOESM2_ESM.zip › README.rst.html]

Global Names Parser


# Global Names Parser

- Brief Intro
- Introduction
- Speed
- Features
- Use Cases
  - Getting the simplest possible canonical form
  - Getting canonical form with infraspecies ranks
  - Normalizing name strings
  - Removing authorships in the middle of the name
  - Figuring out if names are well-formed
  - Creating stable GUIDs for name strings
  - Assembling canonical forms etc. from original spelling
- Dependency Declaration for Java or Scala
- Release Package
  - Requirements
  - Released Files
- Fat Jar
- Command Line Tool and Socket Server
  - Installation on Linux/Mac
  - Installation on Windows
  - Usage of Executable
  - Usage as a Socket Server
  - Usage as a REST API Interface
- Usage as a Library
  - Scala
  - Apache Spark
  - Java
  - Jython
  - R
  - JRuby
- Getting Code for Development
  - Requirements
  - Installation
  - Project Structure
  - Commands
- Docker container
- Usage
- Contributors
- License

# Brief Intro

`gnparser` splits scientific names into their component elements with associated meta information. For example, `"Homo sapiens Linnaeus"` is parsed into human readable information as follows:

| Element | Meaning | Position |
| --- | --- | --- |
| Homo | genus | (0,4) |
| sapiens | specific\_epithet | (5,12) |
| Linnaeus | author | (13,21) |

Try it as a command line tool under Linux/Mac:

```
wget https://github.com/GlobalNamesArchitecture/gnparser/releases/download/release-0.3.3/gnparser-0.3.3.zip
unzip gnparser-0.3.3.zip
sudo rm -rf /opt/gnparser
sudo mv gnparser-0.3.3 /opt/gnparser
sudo rm -f /usr/local/bin/gnparse
sudo ln -s /opt/gnparser/bin/gnparse /usr/local/bin
gnparse name "Homo sapiens Linnaeus"
gnparse --help
```

`gnparser` is also dockerized:

```
docker pull gnames/gnparser
# to run web-server
docker run -p 80:4334 --name gnparser gnames/gnparser web
# to run socket server
docker run -d -p 4334:4334 --name gnparser gnames/gnparser socket
```

Finally, run it right from your SBT console:

```
$ mkdir -p project
$ echo 'sbt.version=0.13.12' > project/build.properties
$ sbt ';set libraryDependencies += "org.globalnames" %% "gnparser" % "0.3.3";console'
scala> import org.globalnames.parser.ScientificNameParser.{instance => scientificNameParser}
scala> scientificNameParser.fromString("Homo sapiens Linnaeus").renderCompactJson
```

# Introduction

Global Names Parser or `gnparser` is a Scala library for breaking up scientific names into their different elements. The elements are classified. It is based on parboiled2 -- a Parsing Expression Grammar (PEG) library. The gnparser project evolved from another PEG-based scientific names parser --biodiversity written in Ruby. Both projects were developed as a part of Global Names Architecture.

Many other parsing algorithms for scientific names use regular expressions. This approach works well for extracting canonical forms in simple cases. However, for complex scientific names and to parse scientific names into all semantic elements regular expressions often fail, unable to overcome the recursive nature of data embedded in names. By contrast, `gnparser` is able to deal with the most complex scientific name strings.

`gnparser` takes a name string like `Drosophila (Sophophora) melanogaster Meigen, 1830` and returns parsed components in JSON format. We supply a description of the output fields as JSON schema. This parser's behavior is defined in its tests and the test file is a good source of information about parser's capabilities, its input and output.

# Speed

Millions of names parsed per hour on a i7-4930K CPU (6 cores, 12 threads, at 3.4 GHz), parser v0.3.1

| Threads | Millions/hr |
| --- | --- |
| 1 | 29.44 |
| 2 | 50.85 |
| 4 | 90.45 |
| 8 | 120.75 |
| 12 | 130.9 |

# Features

- Fast (~8x faster than biodiversity gem), rock solid and elegant
- Extracts all elements from a name, not only canonical forms
- Works with very complex scientific names, including hybrids
- Can be used directly in any language that can call Java -- Scala, Java, R, Python, Ruby etc.
- Can run as a command line application
- Can run as a socket server
- Can run as a web server
- Can be integrated into Apache Spark-based projects
- Can be scaled to many CPUs and computers
- Calculates a stable UUID version 5 ID from the content of a string

# Use Cases

## Getting the simplest possible canonical form

Canonical forms of a scientific name are the latinized components without annotations, authors or dates. They are great for matching names despite alternative spellings. Use the `canonical_form` field from parsing results for this use case.

## Getting canonical form with infraspecies ranks

In botany infraspecific ranks play an important role. Use `canonical_extended` field to preserve them.

## Normalizing name strings

There are many inconsistencies in how scientific names may be written. Use `normalized` field to bring them all to a common form (spelling, spacing, ranks).

## Removing authorships in the middle of the name

Many data administrators store name strings in two columns and split them into "name part" and "authorship part". This practice misses some information when dealing with names like "*Prosthechea cochleata* (L.) W.E.Higgins *var. grandiflora* (Mutel) Christenson". However, if this is the use case, a combination of `canonical_extended` with the authorship from the lowest taxon will do the job. You can also use `--simple` flag for `gnparse` command line tool.

## Figuring out if names are well-formed

If there are problems with parsing a name, parser generates `quality_warning` messages and lowers parsing `quality` of the name. Quality values mean the following:

- `"quality": 1` - No problems were detected
- `"quality": 2` - There were small problems, normalized result should still be good
- `"quality": 3` - There were serious problems with the name, and the final result is rather doubtful
- `"parse": false` - A string could not be recognized as a scientific name

## Creating stable GUIDs for name strings

`gnparser` uses UUID version 5 to generate its `id` field. There is algorithmic 1:1 relationship between the name string and the UUID. Moreover the same algorithm can be used in any popular language to generate the same UUID. Such IDs can be used to globally connect information about name strings or information associated with name-strings.

More information about UUID version 5 can be found in the Global Names blog.

You can also use UUID calculation library in your code as it is shown in Scala example section.

## Assembling canonical forms etc. from original spelling

`gnparser` tries to correct problems with spelling, but sometimes it is important to keep original spelling of the canonical forms or authorships. The `positions` field attaches semantic meaning to every word in the original name string and allows users to create canonical forms or other combinations using the original verbatim spelling of the words. Each element in `positions` contains 3 parts:

1. semantic meaning of a word
2. start position of the word
3. end position of the word

For example `["species", 6, 11]` means that a specific epithet starts at 6th character and ends *before* 11th character of the string.

# Dependency Declaration for Java or Scala

The artifacts for `gnparser` live on Maven Central and can be set as a dependency in following ways:

SBT:

```
libraryDependencies += "org.globalnames" %% "gnparser" % "0.3.3"
```

Maven:

```
<dependency>
    <groupId>org.globalnames</groupId>
    <artifactId>gnparser_2.11</artifactId>
    <version>0.3.3</version>
</dependency>

<dependency>
    <groupId>org.globalnames</groupId>
    <artifactId>gnparser_2.10</artifactId>
    <version>0.3.3</version>
</dependency>
```

# Release Package

Release package should be sufficient for all usages but development. It is not needed for including `gnparser` into Java or Scala code -- declare dependency instead.

## Requirements

Java Run Environment (JRE) version >= 1.6 (>= 1.8 for runner project)

## Released Files

| File | Description |
| --- | --- |
| `gnparser-assembly-0.3.3.jar` | Fat Jar |
| `gnparser-0.3.3.zip` | `Command line tool, web and socket |
|  | server <#command-line-tool-and-socket-server>`\_ |
| `release-0.3.3.zip` | Source code's zip file |
| `release-0.3.3.tar.gz` | Source code's tar file |

# Fat Jar

Sometimes it is beneficial to have a jar that contains everything necessary to run a program. Such a jar would include Scala and all required libraries.

Fat jar for `gnparser` can be found in the current release.

# Command Line Tool and Socket Server

## Installation on Linux/Mac

```
wget https://github.com/GlobalNamesArchitecture/gnparser/releases/download/release-0.3.3/gnparser-0.3.3.zip
unzip gnparser-0.3.3.zip
sudo rm -rf /opt/gnparser
sudo mv gnparser-0.3.3 /opt/gnparser
sudo rm -f /usr/local/bin/gnparse
sudo ln -s /opt/gnparser/bin/gnparse /usr/local/bin
```

## Installation on Windows

1. Download gnparser-0.3.3.zip
2. Extract it to a place where you usually store program files
3. Update your PATH to point to bin subdirectory
4. Now you can use `gnparse` command provided by `gnparse.bat` script from CMD

## Usage of Executable

Note that `gnparse` loads Java run environment every time it is called. As a result parsing one name at a time is **much** slower than parsing many names from a file. When parsing large file expect rates of 6000-9000 name strings per second on one CPU.

To parse one name

```
gnparse name "Parus major Linnaeus, 1788"
```

To parse names from a file (one name per line).

```
gnparse file --input file_with_names.txt [--output output_file.json --threads 8]
```

To see help

```
gnparse --help
```

## Usage as a Socket Server

Use socket (TCP/IP) server when the `gnparser` library cannot be imported directly by a programming language. Setting `--port` is optional, 4334 is the default port.

```
gnparse socket --port 1234
```

To test the socket connection with command line:

```
telnet localhost 1234
```

When you see your telnet prompt, enter a name and press `Enter`.

To use (TCP/IP) socket server in your code find a `socket` library for your language. There is a good chance it is included in the language's core. For example in Ruby it would be:

```
require "socket"
s = TCPSocket.open("0.0.0.0", 1234)
s.puts("Homo sapiens")
s.gets
```

## Usage as a REST API Interface

Use web-server as an HTTP alternative to TCP/IP server. Setting `--port` is optional, 4334 is the default port. To start web server in production mode on http://0.0.0.0:9000

```
gnparse web --port 9000
```

Make sure to CGI-escape name strings for GET requests. An '&' character needs to be converted to '%26'

- `GET /api?q=Aus+bus|Aus+bus+D.+%26+M.,+1870
- `POST /api` with request body of JSON array of strings

# Usage as a Library

Several languages are supported either natively or by running their JVM-based versions. The examples folder provides scientific name parsing code snippets for Scala, Java, Jython, JRuby and R languages.

To avoid declaring multiple dependencies Jython, JRuby and R need a reference gnparser fat-jar.

If you decide to follow examples get the code from the release or clone it from GitHub

## Scala

Scala example is an SBT subproject. To run it execute the command:

```
sbt 'examples/runMain org.globalnames.parser.examples.ParserScala'
```

Calculation of UUID version 5 can be done in the following way:

```
scala> val gen = org.globalnames.UuidGenerator()
scala> gen.generate("Salinator solida")
res0: java.util.UUID = da1a79e5-c16f-5ff7-a925-14c5c7ecdec5
```

## Apache Spark

examples/spark/README.rst describes how to use `gnparser` with Scala or Python in Apache Spark projects.

## Java

Java example is an SBT subproject. To run it execute the command:

```
sbt 'examples/runMain org.globalnames.parser.examples.ParserJava'
```

## Jython

Jython example requires Jython -- a Python language implementation for Java Virtual Machine. Jython distribution should be installed locally according to instructions.

To run it execute the command:

```
java -jar $JYTHON_HOME/jython.jar \
  -Dpython.path=/path/to/gnparser-assembly-0.3.3.jar \
  examples/jython/parser.py
```

(JYTHON\_HOME needs to be defined or replaced by path to Jython jar)

## R

R example requires rJava package to be installed. To run it execute the command:

```
Rscript /opt/gnparser/examples/R/parser.R
```

## JRuby

JRuby example requires JRuby -- a Ruby language implementation for Java Virtual Machine. JRuby distribution should be installed locally according to instructions.

To run it execute the command:

```
jruby -J-classpath /path/to/gnparser-assembly-0.3.3.jar \
  examples/jruby/parser.rb
```

# Getting Code for Development

## Requirements

- Git
- Scala version >= 2.10.6
- Java SDK version >= 1.8.0
- SBT >= 0.13.12

## Installation

```
git clone https://github.com/GlobalNamesArchitecture/gnparser.git
cd gnparser
```

If you decide to participate in `gnparser` development -- fork the repository and submit pull requests of your work.

## Project Structure

The project consists of four parts:

- `parser` contains core routines for parsing input string
- `examples` contains usage samples for some popular programming languages
- `runner` contains code required to run `parser` from a command line as a standalone tool or to run it as a TCP/IP server
- `web` contains a web app and a RESTful interface to `parser`

## Commands

| Command | Description |
| --- | --- |
| `sbt test` | Runs all tests |
| `sbt ++2.10.6 test` | Runs all tests against Scala v2.10.6 |
| `sbt assembly` | Creates fat jars for |
|  | command line and web |
| `sbt stage` | Creates executables for |
|  | command line and web |
| `sbt web/run` | Runs the web server in development mode |

# Docker container

Prebuilt container image can be found on dockerhub

# Usage

To install/update container

```
docker pull gnames/gnparser
```

To run web server

```
docker run -d -p 80:4334 --name gnparser gnames/gnparser web
```

To run socket server

```
docker run -d -p 4334:4334 --name gnparser gnames/gnparser socket
```

# Contributors

- Alexander Myltsev http://myltsev.com alexander-myltsev@github
- Dmitry Mozzherin dimus@github

# License

Released under MIT license
